# Human rights abuses and collective resilience among sex workers in four African countries: a qualitative study

**DOI:** 10.1186/1744-8603-9-33

**Published:** 2013-07-26

**Authors:** Fiona Scorgie, Katie Vasey, Eric Harper, Marlise Richter, Prince Nare, Sian Maseko, Matthew F Chersich

**Affiliations:** 1 Centre for Health Policy (CHP), School of Public Health, Faculty of Health Sciences, University of the Witwatersrand, Johannesburg, South Africa; 2 Maternal, Adolescent and Child Health (MatCH), Department of Obstetrics and Gynaecology, Faculty of Health Sciences, University of the Witwatersrand, Johannesburg, South Africa; 3 Social Science and Health Research, School of Psychology and Psychiatry, Monash University, Melbourne, Australia; 4 African Sex Worker Alliance, London, UK; 5 International Centre for Reproductive Health, Ghent University, Ghent, Belgium; 6 African Centre for Migration & Society, University of the Witwatersrand, Johannesburg, South Africa; 7 Nursing Sciences Department, University of Pretoria, Pretoria, South Africa; 8 Sexual Rights Centre, Bulawayo, Zimbabwe

**Keywords:** Sex work, Prostitution, Violence, Human rights, Resilience, Kenya, South Africa, Uganda, Zimbabwe

## Abstract

**Background:**

Sex work is a criminal offence, virtually throughout Africa. This criminalisation and the intense stigma attached to the profession shapes interactions between sex workers and their clients, family, fellow community members, and societal structures such as the police and social services.

**Methods:**

We explore the impact of violence and related human rights abuses on the lives of sex workers, and how they have responded to these conditions, as individuals and within small collectives. These analyses are based on data from 55 in-depth interviews and 12 focus group discussions with female, male and transgender sex workers in Kenya, South Africa, Uganda and Zimbabwe. Data were collected by sex worker outreach workers trained to conduct qualitative research among their peers.

**Results:**

In describing their experiences of unlawful arrests and detention, violence, extortion, vilification and exclusions, participants present a picture of profound exploitation and repeated human rights violations. This situation has had an extreme impact on the physical, mental and social wellbeing of this population. Overall, the article details the multiple effects of sex work criminalisation on the everyday lives of sex workers and on their social interactions and relationships. Underlying their stories, however, are narratives of resilience and resistance. Sex workers in our study draw on their own individual survival strategies and informal forms of support and very occasionally opt to seek recourse through formal channels. They generally recognize the benefits of unified actions in assisting them to counter risks in their environment and mobilise against human rights violations, but note how the fluctuant and stigmatised nature of their profession often undermines collective action.

**Conclusions:**

While criminal laws urgently need reform, supporting sex work self-organisation and community-building are key interim strategies for safeguarding sex workers’ human rights and improving health outcomes in these communities. If developed at sufficient scale and intensity, sex work organisations could play a critical role in reducing the present harms caused by criminalisation and stigma.

## Background

The majority of countries in the world have punitive laws against sex work [1], and virtually throughout Africa, this occupation is an explicit criminal offence [2]. In practice, however, authorities seldom formally prosecute sex workers under anti-prostitution laws, since these are difficult to prove and enforce. Instead, police invoke municipal by-laws or vague non-criminal legislation to arrest or detain sex workers on charges of “loitering”, “indecent exposure”, “public nuisance” or offences that do not warrant arrest, such as “blocking the pavement”, but often without actually charging them [3-6]. A recent study undertaken in six countries, including Zimbabwe and South Africa, concluded that routine confiscation of condoms by police and use of condoms against them “forces sex workers to make a choice between safeguarding their health and staying safe from police harassment or detention” [7].

The legal status of sex work and the entrenched stigma and discrimination associated with the profession in Africa means that sex workers have historically been viewed as “reservoirs of sexually transmitted disease”, and blamed for the continent’s HIV crisis [8,9]. To address their considerable occupational health risks, researchers and activists in the fields of health and HIV have long argued for a *rights*-*based* approach to sex worker interventions, including the decriminalisation of the profession [10,11]. Indeed, Overs and Hawkins contend that health projects, in particular, are well positioned to document the impact of the law on sex worker human rights and health [12]. For example, in South Africa, concerted efforts by health workers and a non-governmental organisation (Sex Worker Education & Advocacy Taskforce) to document instances of police action interfering with health outreach services for sex workers in Cape Town helped mobilise the Western Cape Department of Health against such police action.

Indifference to the murder of sex workers, alongside a host of other human rights violations, was a key theme and rallying point at an African sex worker conference in Johannesburg in February 2009, the first ever led by sex workers. Several conference participants noted that sex workers in a number of African countries “simply disappear”, and the right to legal redress following human rights abuse is routinely ignored [13]. One outcome of the conference was the formation of a pan-African Alliance to mobilise for sex workers’ rights on the continent and to build support for legal reform in this area.^1^ Among other things, this African Sex Worker Alliance (ASWA) made a commitment to generate credible research on sex workers’ experiences of human rights violations, with sex workers themselves playing an active role in research teams. This article reports on research commissioned by ASWA in 2010 as a direct fulfilment of this commitment.

Using qualitative methods, the research examined the experiences of female, male and transgender sex workers in four African countries (Kenya, Uganda, South Africa and Zimbabwe), with regard to human rights violations perpetrated by police, clients, regular partners, landlords and others involved in the sex industry. In an important addition to existing literature, we ask what strategies sex workers in each country use to cope with or avoid these violations, or indeed to seek formal redress. Drawing on sex workers’ own narratives, we illustrate the combined effects of criminalisation and law enforcement on their everyday lives and social relations, and how this in turn affects their health and wellbeing. Where possible, we highlight differences associated with gender, sexual orientation and migrant status, and draw cross-country comparisons, thereby moving beyond the scope of other published studies on this topic in Africa, which usually report on findings from a single country setting [14,15]. Finally, we explore how sex workers, as individuals or within groups, have responded to their circumstances, developing strategies to cope and mobilize against the multiple violations that emerge as endemic to sex work on the continent.

## Methods

Ethical approval for study activities was granted by the Human Research Ethics Committee (Medical) of the University of the Witwatersrand, South Africa (M10953). Data were collected between December 2010 and January 2011 in six urban sites, namely, Mombasa, Kenya; Kampala, Uganda; and Bulawayo, Zimbabwe; and three sites in South Africa (Hillbrow, Johannesburg, Gauteng Province, and the towns of Musina and Thohoyandou in Limpopo Province). In addition to each of these sites being well-known sex work areas in the countries and provinces studied, they are locations where ASWA has existing operations and networks and where the association intends to deepen its advocacy work. Selection of study sites was therefore warranted as the research would yield valuable information to inform planning of ASWA’s work in these locations. To be included in the study, participants had to be 18 years and older, have had at least one client in the past week, and not be under the influence of drugs or alcohol at the time of recruitment.

### Data collection

Fifty-five in-depth interviews were held, and 12 focus group discussions (each with 5–8 participants, n = 81). Interviews were conducted by female sex worker peer educators (aged 18–40) affiliated to ASWA, who were already familiar with the lives of a diverse range of sex workers, given their experience of advocacy work with these communities. Data collection was coordinated by the principal investigator based in South Africa (FS), who did not travel to study sites owing to limited project funding.

Prior to data collection, interviewers from each country attended a three-day training workshop in South Africa, covering basic research skills and research ethics. During this workshop, interviewers defined the geographical boundaries of their study area and developed a brief synopsis of the local sex worker community, based on their own outreach and peer-education work experience. This synopsis – providing detail on the estimated proportion of sex workers who were migrants, for example, and a breakdown of different sex work settings – was then used to guide recruitment, with the aim being to recruit a sample that would reflect the diversity within the sex worker community in each site.

Interviewers identified a first set of potential participants from their existing peer networks, (thus making it more likely that participants’ actual sex work involvement could be verified). These initial participants were asked by the interviewer at the close of the interview to suggest others who could be approached to take part in the study (i.e. purposive, ‘snowball’ sampling). When approaching potential participants, interviewers verbally checked that all three inclusion criteria for the study (listed above) were met before proceeding with recruitment of participants. Written informed consent was obtained from all participants.

Both individual interviews and focus group discussions (FGDs) were semi-structured, following an interview guide containing mainly open-ended questions. A short demographic questionnaire was administered to all participants at the close of the interviews and FGDs to capture details such as age, education, migration history, other income sources and number of years in the sex industry. During the informed consent process, all FGD participants were asked to respect the confidentiality of other participants in the group and not reveal names of other participants or share what had been discussed in the group with other people.

Interviewers translated the data collection tools into local languages, as required. Most interviews were audio-recorded, although six participants declined recording and for these, hand-written notes were taken. Real names were collected only on informed consent forms; thereafter unique numbers linked participants and interview transcripts. Interviews were held in locations where participant confidentiality and safety could be maximised, such as a participant’s or interviewer’s home, or a private room within a local non-governmental organisation (NGO). Actual names of participants are not reported in this article. In each site, participants were referred to local counselling and legal organisations if such support was required.

### Data management and analysis

Interviewers translated and transcribed the recorded interviews in English. The principal investigator conducted the bulk of the analysis, initially coding interview transcripts thematically, through a process of identifying key themes and developing an interpretive framework for overall analysis of results. This framework was cross-checked by some of the peer interviewers and three other members of the study team, to increase reliability of coding. In both the investigation methods used and in analysis, the authors made deliberate attempts to foreground the perspectives of the sex workers, by allowing the overall narrative of their broader experiences to provide a grounded interpretive context for the analysis of individual themes. In this way, we sought to avoid imposing middle class and western values on the data, or objectifying sex workers as engaging in morally objectionable and demeaning work.

Demographic and sex work characteristics were compared between study groups (women in each of the study sites, and male and transgender participants in all sites). Using Intercooled Stata 11.0 (Stata Corporation, College Station, Texas, USA), chi-square tests detected associations between categorical variables and a one-way ANOVA assessed associations between continuous variables and the study groups (Table 1).

**Table 1:** Socio-demographic and sex work characteristics of female, male and transgender participants in Kenya, Uganda, South Africa and Zimbabwe

**Variable**	**Females Kenya, Mombasa (n = 16)**	**Females Uganda, Kampala (n = 25)**	**Females South Africa**		**Females Zimbabwe, Bulawayo (n = 21)**	**Male and transgender across sites (n = 30)**
			**Hillbrow, Gauteng Province (n = 15)**	**Limpopo Province (n = 29)**		
**Age** mean years (sd)	29 .0 (3.3)	28.6 (7.1)	30.7 (5.6)	26.7 (4.8)	35.9 (7.5)	25.9 (4.6)
**Migration history %** (n)						
Born outside the city	69 (11)	100 (25)	100 (15)	96 (27)	50 (10)	59 (17)
Born outside the country	0 (0)	0 (0)	60 (9)	61 (17)	0 (0)	24 (7)
**Education completed %** (n)						
Primary school	19 (3)	63 (15)	7 (1)	17 (5)	48 (10)	20 (6)
Secondary school	63 (10)	38 (9)	93 (14)	83 (24)	43 (9)	67 (20)
Tertiary	19 (3)	0 (0)	0 (0)	0 (0)	10 (2)	13 (4)
**Mean children** (sd)	2.1 (1.1)	2.5 (1.3)	1.5 (0.9)	1.3 (0.9)	2.4 (1.4)	0.3 (0.6)
**Other income sources %** (n)	6 (1)	20 (5)	33 (5)	69 (20)	52 (11)	23 (7)
**Age began sex work**						
Mean years (sd)	17.6 (3.6)	21.2 (4.4)	25.8 (6.8)	23.1 (4.6)	26.6 (5.0)	19.0 (3.3)
**Years doing sex work %** (n)						
0–2	0 (0)	4 (1)	27 (4)	41 (12)	0 (0)	33 (10)
3–9	38 (6)	68 (17)	67 (10)	52 (15)	57 (12)	27 (8)
≥10	63 (10)	28 (7)	7 (1)	7 (2)	43 (9)	40 (12)
Current workplace(s)*% (n)						
Hotel	44 (7)	36 (9)	20 (3)	14 (4)	24 (5)	43 (13)
Bar or club	56 (9)	72 (18)	60 (9)	24 (7)	57 (12)	67 (20)
Brothel	50 (8)	48 (12)	47 (7)	3 (1)	0 (0)	17 (5)
Street	44 (7)	96 (24)	20 (3)	83 (24)	62 (13)	67 (20)
Home	50 (8)	20 (5)	0 (0)	21 (6)	38 (8)	47 (14)

P values assessing associations between study groups (table columns) and characteristics (table rows) all <0.05. *Multiple-response question; *sd* standard deviation.

## Results

### Study participants

Interviews were held with female (106), male (26) and transgender (4) sex workers (see Table 1). Females in Zimbabwe were aged a mean 35.9 years, while other females were an average 28.4 years, similar across sites. Male and transgender participants were younger, with 63% of this group between 18 and 25 years. Participants had mostly entered the sex industry for economic survival, with women in Kenya beginning sex work as young as 17.6 years on average and men at 19 years, younger than mean sex work onset in Hillbrow (25.6 years) and Zimbabwe (26.6 years). All female participants interviewed in Kenya, Uganda and Zimbabwe had been born in their home country, though predominately they now worked outside the city of their birth. By contrast, around 60% of participants in South Africa, both in Hillbrow and the Limpopo province sites, and a quarter of the male and transgender sex workers, were cross-border migrants.

A variety of sex work settings were reported. Female participants in Hillbrow were overwhelmingly bar- and brothel-based, while street-based sex workers formed the largest group of participants in Kampala, Limpopo and Bulawayo. Once a client had been secured, participants across sites reported having sex in the ‘bush’, in a nearby lodge or hotel, at the client’s home, or at their own home.

Although many were driven to sell sex to escape severe poverty and unemployment, several sex workers reported that the work was attractive because it had given them financial independence and the ability to improve their economic circumstances. One participant saw it as an advantage that money from sex work “comes right there and then, unlike other jobs where the money will come late” (30 year old male, Bulawayo). Another stated: “I manage my own business – my money is not taxed” (34 year old female, Hillbrow). Many sex workers, especially women, used their income to support family members and pay school fees. The vast majority of women had children (94%, 100/106), and around a quarter had three or more (25/106). A few used money from sex work to create alternative income-earning ventures, such as buying sewing machines or additional land to rent, or opening a bar. A half to two thirds of women in Limpopo and Zimbabwe reported this, although in many instances these income sources appeared quite erratic. Under a third of women elsewhere and of males and transgender participants had alternative income.

For many of our participants, “exiting” sex work was not seen as a viable option, as many had limited education and thus few alternatives to earn income.^2^ In South Africa and Kenya, a large proportion of female participants had completed secondary school (93% Hillbrow, 83% Limpopo and 81% Kenya). By contrast, most of the Ugandan (63%) and Zimbabwean (48%) women had only attended primary school. In short, despite its considerable drawbacks – as catalogued below – there are real advantages to engaging in this work that may not be available elsewhere, such as managing one’s own working hours, ready cash, earning higher wages than in other available employment [16,17] and being able to assert some measure of independence over one’s working environment.

### Ownership and violence: client and sex worker interactions

The narratives of most of the sex workers we interviewed were dominated by descriptions of poor treatment at the hands of clients. A pattern emerged in which clients, by paying for sex, behaved as if they had full ‘ownership’ over the sex worker, and payment for services was seen to offer clients ‘*carte blanche*’ to do as they wish. This included ignoring sex workers when they made requests, for example, regarding condom use or preferred sex positions, or if they indicated they were in pain.

*“You can tell the client to stop and he says, ‘didn’t I buy you with my money? I have to get my money’s worth first.’ For you don’t appear to him like a human being. So he mistreats you…when he leaves he may hit you on your face and it gets swollen.”* (Female focus group, Kampala)

*“A man came to me and approached me as a client, then we go to the bush and he sleep with me in a hard way. When I say ‘you are hurting me’, he said ‘I pay you, so don’t tell me shit.’”* (25 year old female, Thohoyandou)

Clients also frequently tricked the sex worker into accepting a smaller payment or refused to pay for services at all:

*“We meet clients who are so rough. Sometimes some pretend they want a short-time so when you are done, they refuse to pay.”* (47 year old female, Bulawayo)

The illegal nature of sex work in all study sites was something that clients frequently took advantage of, using it to legitimate non-payment for services. This attitude was invoked even more readily with street-based sex workers, with illegal migrants and - in countries where same-sex relationships are criminalised (Kenya, Zimbabwe and Uganda) - with male and transgender sex workers:

*“Sometimes a man will take you and after fucking, he says, ‘You are gay, where can you report me? I’m not paying you and you can do nothing about it.”* (Male focus group, Kampala)

In all sites, virtually all sex workers reported being physically beaten by clients or threatened with firearms, with many having experienced this repeatedly. Several pointed out that accompanying a client to his home, as opposed to meeting him in a ‘neutral’ space, such as a room in a lodge or the sex worker’s own living space, increases their vulnerability to abuse. This principle was most evident in descriptions by both male and female sex workers of an alarming pattern of gang rape by clients. After agreeing to have sex with a client and arriving at the venue, there would be several other men waiting to have sex with them as well. A woman in Uganda related one such incident:

*“A man can come and tell you that, ‘I want to spend a night with you.’ …So the man tells you that he stays alone; sometimes he even shows you the house keys and you go with him. When you reach there he opens the door and you enter or even you sleep with him and then he pretends as if he is going out to bath and there comes in others, like four of them and they all have sex with you.”* (28 year old female, Kampala)

Having experienced several such incidents in the past, this participant asked one of the men “why do you do things like that?” His response revealed that the strategy was one commonly employed by poor men who could not afford to individually pay for the services of sex workers. He explained:

*“We collect money amongst ourselves. So then one comes and negotiates with a woman and he takes you. After he finishes you he calls the rest of the others.”* (cited by 28 year old female, Kampala)

This practice further illustrates the extent to which clients generally assume “ownership” of sex workers, objectifying them as a commodity that can be bought and then shared among friends.

### Between a rock and hard place: criminal laws and police impunity

While gang rape perpetrated by clients was distressingly common, gang rape by police and related authorities was also reported across sites and in forms that were remarkably similar:

*“There was this time when I was arrested by six policemen. They afterwards demanded sex from me. One of them threatened to stab me if I refused. I ended up having sex with all of them and the experience was so painful.”* (26 year old male, Mombasa)

*“They were policemen. There’s a car park next to the flat and they took me there and they took turns.”* (26 year old female, Bulawayo)

Equally common was physical abuse by police, often taking extreme forms. Virtually all sex workers interviewed had experienced being beaten and assaulted by police at some point in their working lives.

*“I remember when the riot police once picked us up, we were severely beaten, insulted and mocked by the police, I won’t forget that day. I was badly injured. My child asked what had happened to me and I had to lie and say I was attacked by robbers.”* (48 year old female, Bulawayo)

With male and transgender sex workers, this police abuse often took on an added dimension of homophobia. A 29 year old transgender sex worker in Uganda recounted a particularly humiliating experience:

“They [the police] arrested me and undressed me and asked me whether I was a woman or man. They beat me and detained me in prison.”

Similarly, cross-border migrants also reported experiences of being singled out and denigrated by police – particularly in the two South African sites:

*“Police are always complaining that we are not supposed to come here and do this business. They call us names – ‘makwerekwere’ (derogatory term for foreigner in South African slang). They arrested us and sprayed us with pepper spray inside… that big van. People were vomiting and coughing. Me, they sprayed into my eyes, saying ‘go back to your country.’”* (36 year old female, Hillbrow)

About half the participants described being arrested by police, but frequently they were either released before arrival at the police station, or were taken there but not formally charged or detained. In the east African sites, however, some sex workers reported being arrested and detained for fairly long periods of time, ranging between one week and six months.

In all study sites, bribery, blackmail and sexual abuse by police was described as part-and-parcel of the daily working life of sex workers, a situation that was seen as being legitimised by the criminalisation of sex work. Many participants responded ‘all the time’ when asked “in the past year, have you been forced to pay bribes to the police or anyone else?” Unsurprisingly, routine police violations of the rights of sex workers, their lived experiences of criminalisation, and the consequent impunity of individual police officers from any form of punishment, have instilled a deep sense of futility among sex workers about being protected by the police or the value of reporting abuses to them. Most participants were adamant that they would not seek redress for the violations they had experienced.

*“When a man rapes you, beats you or uses you, you cannot go and report him because you are a sex worker, though other people who are not sex workers can go and report. I just keep quiet and die with my pain… When you go to report you will be asked, ‘What were you doing?’ and you will be charged with prostitution, so the laws do not favour us and we cannot report cases.”* (33 year old female, Kampala)

In the very few instances where sex workers had reported violations to the police, the response was usually negative and generated further trauma:

*“The day I was raped I went to report to the police, but the harassment I got there almost made me faint. The police wanted me to explain every detail from the rape to the screams that I made. Another officer asked how a prostitute like me could be raped as I was used to all sizes. He told me in fact that man really spared me. He could have tested my ass too. He ended asking me if my ass is already opened. Never will I again go to report a case. I’d rather die.”* (32 year old female, Mombasa)

### Landlords, brothel managers and regular partners

While clients and police are perhaps the most visible role-players in the working environment of sex workers, a wide range of people, including landlords, hotel and bar staff, security guards and brothel owners, are either centrally involved in the sex trade or operate on its fringes. Our research found that these individuals frequently take advantage of sex workers’ vulnerable position and the illegality of sex work to extort money or sex.

*“One day I was harassed by a client and when I told the bar manager he demanded sex so that he can help me. My landlord also demanded sex because I could not afford rent.”* (25 year old female, Mombasa)

Indeed, landlords demanding sex in exchange for accommodation or basic services was a common experience across study sites, and particularly true for female sex workers who tended to live together in brothels or hotels (more so than male and transgender participants), and were therefore vulnerable to these sorts of demands. Just over a quarter of participants reported working from brothels; they recounted the fraught nature of negotiating terms with a brothel manager in a generally exploitative relationship:

*“When you go with a client, the brothel manager takes 50% of your money. Since I am the one who toils for this money, I thought the manager would be taking a smaller percentage. Even when a client goes without paying me, I still have to pay the manager.”* (26 year old female, Kampala)

More than a third of males (41%) and females (37%) reported having a regular partner. Most described these relationships as abusive, characterised by frequent verbal bullying, physical violence or rejection on account of their sex worker status, even if initially their profession had not seemed problematic to a new partner.

*“My boyfriend harasses me because I am a sex worker and he demands sex, and if I refuse he rapes me. There was a time when he raped my child when I was at work. And I cannot get rid of him because if I try to chase him away he says that he will try his best to expose me.”* (Female, 18 years, Mombasa)

Others had made deliberate decisions to eschew relationships with regular partners altogether, on account of these risks.

### Our daily bread: abuse from family and community members

Many participants expressed fears of gossip and vilification by neighbours, and consequently tried to conceal their sex worker status from community members. Sex workers working on the streets, in particular, described being frequently shouted at by passers-by, laughed at, called names, and accused of ‘stealing men’ or of being HIV positive. A Limpopo-based participant explained the source of her neighbours’ deep suspicion of sex workers as stemming from fear that they would sleep with their husbands:

*“Married women, they are a big problem. They hate us.”* (Female focus group, Musina)

A Kenyan woman, aged 32, recalled an incident where these suspicions were acted out in a particularly humiliating way:

“One day I was at a club when a neighbour’s husband joined the table [where] I was seated. Someone saw us chatting and called his wife. The wife brought a few other neighbours and protested at the club. They beat me up and took me to the toilet. There, they pushed my head inside a dirty sink to drink the sewer. It was horrible. The husband ran, instead of telling them that we were not doing anything bad, just talking. Back at home, the landlord told me that I could no longer reside in his house as the women were furious with me because I would steal their husbands.”

As this example (and many others like it) suggests, it is difficult for female sex workers to forge bonds of solidarity with other women their communities. Women in these contexts are also easy targets of blame, with men for the most part being allowed to escape accountability for their actions. To counter this kind of treatment by neighbours and other community members, a Ugandan participant said that the sex workers she knew had made a deliberate decision to sell sex only to men from other neighbourhoods (female focus group, Kampala).

Some participants recalled being insulted in public by children, an especially painful affront, given that in many African communities it is a widespread social norm that children demonstrate respect for older generations. In Limpopo, a sex worker described how she had been taunted by children:

*“It happens to me waiting for a taxi: small kids about 10-years old asking me to have sex, saying ‘I have 10 rand [about 75 pence].’ I refuse and they start to shout, ‘You sleep with our fathers at night!’”* (Female focus group, Musina)

Common terms of abuse included: ‘bitch’, ‘whore’, ‘AIDS-man’, and insulting references to dogs:

[They say] *‘You’re just a prostitute and prostitutes are like dogs*.*’* (26 year old female, Kampala)

The notion that sex workers were not perceived to be “human” emerged strongly in all study sites, but particularly in east Africa.

*“[People in the community say] I’m not a human being … I am just useless, spoilt and that’s the end of me. They can’t allow me to spoil others. When they see my child they say ‘that’s a prostitute’s child, look at it, child of a prostitute.’”* (24 year old female, Kampala)

Aside from these more overt forms of stigmatisation and abuse, sex workers also reported being socially excluded by family, neighbours, and community members. Virtually all examples of social exclusion were provided by participants in the east African countries; Zimbabwean and South African participants tended to give less emphasis to this theme. If someone in the community hosted a party or organised a traditional ceremony, everyone would be invited, but women known to be sex workers would be deliberately overlooked. Similar discrimination even occurred in community-based initiatives, such as projects to support children orphaned by AIDS:

*“We also have children whose fathers died, but we cannot go to those organisations for support. They say they cannot deal with sex workers; they want decent people.”* (Female focus group, Kampala)

In Uganda, sex workers described being excluded from money-lending projects, even those dedicated to “developing women.” One explained:

*“When a sex worker goes to them to ask for a loan so that she may start up a business which she will manage during the day, and at night go to do sex work so that she may pay back their money, they refuse, because she is a sex worker.”* (Female focus group, Kampala)

Social stigma associated with sex work in this country was so all-encompassing, that it even negated the not-insubstantial social and financial contributions made by individual sex workers to their families and communities. In the following exchange, a 32 year-old Ugandan sex worker expresses her frustration with this situation:

“I still have a challenge with the people in the community. People we live with, they don’t want to associate with us. If you give them all you can, they only keep on saying ‘you are a prostitute,’ no matter what good things you do for them. My whole family depends on me alone, even when a person in my family dies, I have to meet all the expenses. Yet people still call me a prostitute.”

Several participants had been expelled from their homes by parents, other family members or community leaders, on account of the work they do:

*“My family threatens me. One time I was chased from home after they realised what I was doing, being a sex worker and a male and I stayed away for some time.”* (25 year old male, Kampala)

As this last participant intimates, for male sex workers there is often a double stigma attached to being involved in sex work and being identified as gay. Similar experiences of stigmatisation and ostracism were reported by a 26-year old transgender sex worker in Mombasa:

“Muslim elders and the community at large… they threatened to stone me to death if I go back there [home]. Now I keep a distance from my village and my people.”

This sex worker’s experience of being ostracised by religious elders was not uncommon. Fierce rejection by religious institutions often took expression in appalling forms of abuse, much of this linked to their religious objections to homosexuality. A male sex worker, aged 26 and from Mombasa recalled:

“Religious leaders have neglected me and do not want to be associated with me. This has left me very lonely. They always point fingers at me. The pastor even made me stand before the congregation and after telling them that I was a sex worker, he excommunicated me from the congregation. I will never forget how all those people shouted at me, calling me all sorts of names. I was really traumatized by this incident, to the extent that I tried to commit suicide.”

### Agency, survival and resistance

Although the study did not set out to capture the long-term psychological consequences of experiencing such high levels of recurrent abuse, public humiliation and the ever-present risk of violence, this theme emerged quite strongly in our participants’ narratives. After reeling off a list of violations she had experienced in her work, for example, one Ugandan woman could go no further, describing these memories as “very heavy”.

To cope with the challenges of working under extreme stress and living with the deep levels of trauma that result, many sex workers described developing what can only be termed ‘survival strategies’. Some attempted to be discerning in their selection of clients, hoping that by scrutinising them carefully, they would choose only non-violent ones:

*“…some people look strange, so I don’t pick those who look strange.”* (35 year old female, Bulawayo)

This strategy, of course, only worked some of the time, and many could not afford financially to be selective with clients. Others developed ways to trick, avoid or even confront the police. For example, when military police in Uganda rounded up a group of sex workers and raped them,

*“…the only one who survived played a trick and started vomiting, saying that she was very sick and no military officer could rape a person who was vomiting.”* (32 year old female, Kampala)

In some cases, sex workers literally fought back against their abusers and succeeded in overpowering them. A focus group participant in Bulawayo recalled her response when assaulted by a police officer:

“Unfortunately one of the policemen who had arrested me got angry because I refused to bribe them. He took me to another room and started slapping me. Later he took his stick and started beating me up. I don’t know what got into me that moment but I shot up at him and beat him nearly to death. When I came back to my senses his arm was broken, he no longer had his shirt on and the room was upside down. I still can’t believe they let me go after that.”

Such acts of direct resistance – particularly those that enabled sex workers to escape further violence – seemed rare, however. To avoid arrest or to escape dangerous situations, sex workers commonly gave in to police officers’ demands for bribes, whether involving money or sex. Importantly, sex workers characterised these actions of the police as “theft” or “robbery” rather than bribery, a strategic reframing that may in itself serve as an act of resistance.

As described above, reporting incidents to the police was seldom considered a viable option. But not all sex workers who had been violated accepted that they had no recourse to the law. A woman from Thohoyandou, Limpopo, showed that standing up for one’s rights could have a positive outcome. After agreeing on a rate and spending the night with a client, he refused to pay.

*“Then I told him that, ‘I will get you arrested.’ I took his car keys and went to the police, I told the police that I had an agreement with someone who promised to give me R500 [about 33 pounds] but he did not. So the police said I must wait until he comes to the police station. Within 20 minutes he arrived, then he told the police we agreed on R200 and now I want more. Then the police asked me if he used a condom and I said no. Then the police said he must give me my money in full and I must give him the car keys.”* (21 year old female, Thohoyandou)

Occasionally, sex workers used innovative approaches to confront their would-be abusers, by actively taking control of the situation and demanding respect. This same participant explained:

*“On the 31*^*st*^*of December l called some of my clients for a party at my house and I explained to them the bad things that they do to me and give me stress. Some are now more than my clients; they are now like my friends or cousins. One day I met one of them at the shopping centre then he just greeted me and gave me R20 [about 1.30 pounds] to buy drinks.”* (21 year old female, Thohoyandou)

One of the few respondents who reported having had no experiences of police harassment or abuse was a male sex worker aged 24 from Zimbabwe whose clients were women, a factor that for him explained why he was, effectively, ‘invisible’ to the police.

“With matters regarding sex work I have not had any problems with the police. The reason for this is probably that I’m not gay and the police don’t know much about male sex workers who are straight. So whenever I bump into a police officer while looking for clients, they don’t even give me problems because they would be thinking that I am doing other kinds of business, not sex work. It’s really a good thing that the police are ignorant about this kind of job.”

This decision to remain underground and operate covertly as a sex worker was not mentioned by other participants, but may well be a strategy to avoid police targeting that is used more widely in the study sites by sex workers who were invisible even to our interviewers.

### Building resilience through collective action

In addition to individual coping strategies, many participants mentioned the importance of collective action, for example, in building friendships and solidarity with fellow sex workers. Several highlighted the need for unity among sex workers:

*“If we are united, we can do whatever is possible to ensure that people don’t despise us.”* (28 year old female, Kampala)

Participants also discussed the notion of being proactive and participating more fully and more visibly in broader community work, implicitly rejecting deliberate efforts to exclude them. Mobilising sex workers to initiate their own projects was also felt important,

*“…because nobody will come from outside to help sex workers. We as sex workers should lobby for our own health facility which is well equipped and furnished with drugs. This will improve our health and welfare, such that people look at us as people who are well off. Together we can.”* (Female focus group, Kampala)

Some sex workers in Uganda reported having started up savings groups and other microfinance schemes among themselves. While essentially a positive initiative, as it had encouraged disciplined saving among sex workers, there were challenges in the timely payment of contributions and, in some instances, in

*“…the treasurer eating the money and promising to pay when her client comes.”* (Female focus group, Kampala).

Aside from the financial and potentially political importance of initiatives to bring sex workers together, these were also described as important for psychological reasons:

*“When we sex workers meet together, we discuss many issues and advise one another. We comfort ourselves and come with good ideas, which can help us and this makes us feel like we are also human beings and relieves us from stress.”* (Female focus group, Kampala)

The high mobility of sex workers, however, may hamper their efforts to form sustainable groups or collectives. Several participants pointed out that while they would like such groups to form in their areas, the transient nature of sex workers lives would likely undermine the continuity and longevity of such groups. Additional challenges were posed by the threat – or reality – of police harassment:

*“It is difficult for us to have groups in my place because when police find out that there is a group of sex workers they can beat us and arrest us.”* (Female focus group, Kampala)

Participants emphasised the importance of support from local NGOs dedicated to supporting sex workers, where these existed. This support could take many forms, from working together in advocacy, facilitating sex workers to form their own groups, improving legal redress, supplying bail for arrested sex workers, to offering training in human rights defence and practical skills building. A woman in Bulawayo and another in Hillbrow explained how sex worker NGOs help them to assert their rights:

*“We are being assisted by the Sexual Rights Centre, they hold workshops for us and it is through them that I have come to know my rights.”* (42 year old female, Bulawayo)

*“[When experiencing abuse] I call the Sisonke member to ask them what I am supposed to do, because some of us, we don’t know our rights*.*”* (34 year old female, Hillbrow)

Collective empowerment of the sex worker community through building knowledge of their rights cannot be overestimated. When sex workers have been trained and supported in this way, it can foster the confidence and resilience of individual sex workers to take control of dangerous situations. Revealing a potentially useful strategy, a sex worker from Hillbrow claimed that he confronts the police and states his rights, which usually results in them leaving him alone: “Most of the cops, they always say I am crazy, because I always tell them about my rights.” (25 year old male, Hillbrow).

In addition to establishing sex worker groups and support organisations, participants identified other potentially empowering interventions. These included training to improve their sex work skills (as one woman in a focus group in Kampala put it, to enable them to “do our work well”); English language classes, adult literacy training and broader skills training for sex workers themselves, “because most sex workers, we did not get good education” (Female, 28 years, Mombasa). They also appealed for religious institutions to learn more about the context of sex workers’ lives and thus re-evaluate their attitudes towards sex workers, and for training of police and health workers to make them aware of the trauma caused by their actions. Similarly, others called for support to

*“…get connected with the local leaders and the police so that the give us peace and they stop harassing us.”* (Female focus group, Kampala)

Finally, they made a strong request for “a better place” and “a building” (female focus group, Musina) where street-based sex workers could live and work in safety.

## Discussion

Sex workers in the four African countries face diverse forms of violence from all sections of society with whom they interact, from clients, police, landlords and brothel owners, to family and community members. Acting in concert, such forces help to legitimize inequality, alienation, and powerlessness, raising crucial questions concerning the human rights of sex workers in southern and eastern Africa, as elsewhere in the world. Though the socio-political context varies considerably in the four study countries, the experiences of sex workers were largely uniform across sites. Put another way, the experiences of sex workers as documented in this study reflect an alignment and interaction of state, law, social discourse, institutions, philosophies and general public opinions. Though varying somewhat in their exact expression between sites, these entities coalesce around the criminalisation, sexual moralism and associated stigmatisation of sex work. Such attitudes are fuelled by the rise in conservative, religious fundamentalism that is evident in many parts of Africa, and which condemns sex work - alongside homosexuality - and any rights-based discourse associated with them, as socially deviant. Similarly, clients’ treatment of sex workers as objects to be bought and “owned” may be a reflection of a broader gender conservatism in these societies, where women are regarded in general as property of men, with few rights to individual autonomy and freedom.

Consequently, individual sex workers are highly vulnerable to human rights violations, yet have very limited resources or possibilities to challenge perpetrators, or to seek justice and legal compensation. This in turn exacerbates their marginalisation and social exclusion, and limits the options available to them for rising above their precarious circumstances. Those already on the margins of society - such as cross-border migrants, refugees or men who have sex with men - are further isolated and stigmatised when they take up sex work, and enjoy even less protection from the law [18-20].

Practically, the everyday circumstances described by sex workers impact profoundly upon their physical, mental and social wellbeing. Aside from the more direct forms of violence meted out by police or clients, and the humiliation caused by name-calling and “whore stigma” [21], sex workers routinely experience more ‘indirect’ forms of violence. These take the form of deliberate exclusion from social gatherings and community initiatives, which for female sex workers often means remaining outside of the conventional bonds of solidarity among women, from which they might otherwise benefit. The even higher levels of humiliation and abuse extended to male and transgender sex workers culminate in a process of profound ‘othering’, where they are treated as objects decisively beyond the boundaries of decent society.

Both direct and indirect forms of violence impinge upon the space within which sex workers live and work, diminishing the control they have over this space and the way they can move and act within it. Violence, human rights violations and the ensuing trauma can thus be understood as ultimately stripping the person of a safe mental or physical space within which to retreat. This draws attention to the larger problematic of the interconnections of place, culture, and health [22,23] and the importance of understanding the marginalisation and violence so commonly experienced by individual sex workers in terms of the spatial dimensions of sex work more broadly [24].

From a public health perspective, the criminalisation of sex work constrains efforts to reach the population with required health services, and ultimately, structural and legal reforms are necessary [25]. These should align public health and human rights goals, and promote protection of sex workers from violence, HIV and other social harms - goals that would be best achieved in a decriminalised legal framework [26]. Only in South Africa has the possible decriminalisation of adult prostitution been raised in parliamentary discussions, triggering broad public debate, although actual law reform seems a long way off [2].

The study has some limitations, stemming mostly from difficulties inherent in examining such a highly sensitive topic. Firstly, participants’ awareness of the risk of exposure to the authorities may have restricted the openness with which they shared information with the researchers. Indeed, some of the interviews were marked by troubling silences, with participants appearing to either conceal information or limit the degree of detail provided. Secondly, ‘snowball’ sampling tends to recruit sex workers that are more visible, cooperative, and interested in participating in research, rather than capturing a truly representative sample [27]. Although efforts were made to select a heterogeneous sample that reflected the diversity of the sex worker population in each site, it is unclear whether this was actually achieved. It is also possible that the demographic composition of the interviewers (all black African females) influenced sample selection, leading to under-representation of male and transgender participants, and those of other races.

Thirdly, most interviewers had little or no previous research experience, since their background was essentially that of peer education and outreach work. The study budget allowed for only a three-day research methods training workshop, which perforce could only cover the key principles of research and research ethics. Comprehensive training in methods and interviewing skills was not possible. Thus, while there are distinct advantages to using peers to collect data (for example, trust may be established more easily than with non-peer interviewers, potentially yielding richer data), this approach poses considerable challenges. It also cannot be assumed that peers necessarily make good interviewers. By virtue of inhabiting the same local context as the participants, peer interviewers tend not to interrogate shared meanings as much as outsider researchers might. In this instance, interviewers more experienced at probing may have obtained more detailed information, thus enabling a more nuanced assessment of differences across sites and between sex work sub-groups. Peers may also have a vested interest in concealing certain information that could potentially reflect badly on their community or reveal hidden knowledge to outsiders. That participants are asked to share information and experiences with peer interviewers who may encounter them in their local context long after the research has ended, further raises the potential for confidentiality to be breached, or for participants to conceal information, fearing such breaches. By placing full responsibility for data collection and transcription in their hands, however, the peer interviewers in this project were able to learn valuable research skills, broaden their understanding of the experiences of sex workers in their area, and develop links for future advocacy and capacity-building possibilities.

## Conclusion

Our study complements other work done on the continent that has documented sex worker agency in response to extreme human rights violations, and their choice and adoption of proactive livelihood strategies in the face of broader adversity and structural violence [28-32]. It is significant that sex workers in our study draw mostly on informal, *ad hoc* means of support within the broader sex work community. In the short term, interventions building on sex workers’ individual and collective agency and resilience could help to reduce the kinds of human rights violations described here, and their impact on sex worker communities. Examples of successful efforts include structural interventions to tackle violence through building sex worker collectivisation and solidarity in many parts of the world [33], but particularly in India [34]. Given that sex workers commonly face extreme harassment and violence in both living and work spaces – as described above – the creation of ‘safe spaces’, in the form of drop-in centres and clinics, have become favoured interventions. In these ‘safe spaces’, external rules do not apply and sex workers are free to mingle with one another on their own terms. They thus serve to counteract somewhat the disempowering nature of their working environment [34]. There are encouraging signs that such interventions are increasingly being planned at sufficient scale and intensity in Africa, based on evidence and with the involvement of sex workers.

Sex workers’ fears of being exposed, which entrenches a deep reluctance to be public about their experiences and therefore to claim their rights, is slowly beginning to change, as sex workers across the continent are increasingly finding their voices and telling their stories.^3^ Alongside text messaging, video productions, magazines and internet blogs, dance and drama have been used to give sex workers an opportunity to narrate their experiences, share survival strategies and articulate their demands for recognition. These innovative approaches have the potential to help build collective resilience, reduce isolation, and present a humanised and positive public image of sex workers.

In closing, there is an important role for scholarship that catalogues how sex workers on the continent experience and respond to human rights violations. Such documentation must be used to guide the direction of local advocacy efforts and support the development of context-specific, evidence-based interventions to reduce violence against sex workers. Together with such contributions, it is hoped that this research will add further impetus to arguments for much-needed legal reforms [35,36]. For too long, the criminal status of sex work has been used to legitimize any kind of mistreatment of sex workers, and an apparent widespread acceptance in the general public that such conduct is justifiable. Tangible change in this area is long overdue.

## Endnotes

^1^See: http://africansexworkeralliance.org

^2^This information is important to state, since some organisations and funders design interventions solely around the goal of ‘rehabilitating’ sex workers or enabling them to ‘exit’ the industry (Arnott and Crago 2009).

^3^See: http://africansexworkeralliance.org/blog

## Competing interests

The authors declare that they have no competing interests.

## Authors’ contributions

FS led the design of the study, trained interviewers, coordinated data collection, performed the thematic analysis, wrote the first draft and edited subsequent drafts of the manuscript. KV helped draft and edit the manuscript. EH conceived of the study, participated in its design and coordination, and helped draft the manuscript. MR assisted in the training of interviewers, helped analyse the data and draft the manuscript. PN and SM each supervised data collection in one study site and helped draft the manuscript. MFC gave input into study design, data collection and analysis, helped draft the manuscript, and coordinated overall editing. All authors read, gave substantial input and approved the final manuscript.
